# Workers’ physical activity data contribute to estimating maximal oxygen consumption: a questionnaire study to concurrently assess workers’ sedentary behavior and cardiorespiratory fitness

**DOI:** 10.1186/s12889-019-8067-4

**Published:** 2020-01-08

**Authors:** Tomoaki Matsuo, Rina So, Masaya Takahashi

**Affiliations:** 1 Occupational Epidemiology Research Group, National Institute of Occupational Safety and Health, Japan, 6-21-1, Nagao, Tama-ku, Kawasaki, 214-8585 Japan; 2 Research Center for Overwork-Related Disorders, National Institute of Occupational Safety and Health, Japan, 6-21-1, Nagao, Tama-ku, Kawasaki, 214-8585 Japan

**Keywords:** Occupational health, Physical fitness, Reliability, Sitting time, Validity

## Abstract

**Background:**

Sedentary behavior (SB) and cardiorespiratory fitness (CRF) are important issues in occupational health. Developing a questionnaire to concurrently assess workers’ SB and CRF could fundamentally improve epidemiological research. The Worker’s Living Activity-time Questionnaire (WLAQ) was developed previously to assess workers’ sitting time. WLAQ can be modified to evaluate workers’ CRF if additional physical activity (PA) data such as PA frequency, duration, and intensity are collected.

**Methods:**

A total of 198 working adults (93 women and 105 men; age, 30–60 years) completed anthropometric measurements, a treadmill exercise test for measuring maximal oxygen consumption (VO_2max_), and modified WLAQ (m-WLAQ, which included questions about PA data additional to the original questions). Multiple regression analyses were performed to develop prediction equations for VO_2max_. The generated models were cross-validated using the predicted residual error sum of squares method. Among the participants, the data of 97 participants who completed m-WLAQ twice after a 1-week interval were used to calculate intraclass correlation coefficient (*ICC*) for the test–retest reliability analyses.

**Results:**

Age (*r* = − 0.29), sex (*r* = 0.48), body mass index (BMI, *r* = − 0.20), total sitting time (*r* = − 0.15), and PA score (total points for PA data, *r* = 0.47) were significantly correlated with VO_2max_. The models that included age, sex, and BMI accounted for 43% of the variance in measured VO_2max_ [standard error of the estimate (*SEE*) = 5.04 ml·kg^− 1^·min^− 1^]. These percentages increased to 59% when the PA score was included in the models (*SEE* = 4.29 ml·kg^− 1^·min^− 1^). Cross-validation analyses demonstrated good stability of the VO_2max_ prediction models, while systematic underestimation and overestimation of VO_2max_ were observed in individuals with high and low fitness, respectively. The *ICC* of the PA score was 0.87 (0.82–0.91), indicating excellent reliability.

**Conclusions:**

The PA score obtained using m-WLAQ, rather than sitting time, correlated well with measured VO_2max_. The equation model that included the PA score as well as age, sex, and BMI had a favorable validity for estimating VO_2max_. Thus, m-WLAQ can be a useful questionnaire to concurrently assess workers’ SB and CRF, which makes it a reasonable resource for future epidemiological surveys on occupational health.

## Background

Many studies have shown that excessive sedentary behavior (SB) increases disease risk [1, 2]. In this mechanized society, workers are particularly likely to be placed in sedentary situations in the workplace [3, 4]. Therefore, workers’ SB is an important risk factor for occupational health. Similarly, cardiorespiratory fitness (CRF) is a conventional health issue because many studies have shown that low CRF level is strongly associated with increased disease and mortality risks [5]. Recent studies have indicated that midlife CRF plays a role in health-related incidents in later life such as the development of severe diseases [6], increased healthcare costs [7], and decreased longevity [8].

Thus, from the perspective of preventative medicine, both SB and CRF in working adults are key factors in occupational health. However, the relationship between workers’ SB and CRF as well as their interaction effects on disease risk has rarely been investigated in epidemiological surveys. One crucial reason would be inherent in the assessment methodology. The gold standard methods for SB and CRF assessment, such as thigh-worn inclinometer including the activPAL [9] for SB and maximal oxygen consumption (VO_2max_) for CRF, require relatively high cost and considerable time; thus, they have a practical disadvantage for a population-based assessment. In epidemiological surveys, subjective measures such as questionnaires remain useful because they are more cost-effective and present a lower participant burden [10], although the key limitation of the questionnaire method is poor validity. To the best of our knowledge, validated questionnaires to simultaneously assess workers’ SB and CRF have not been proposed.

The Worker’s Living Activity-time Questionnaire (WLAQ) was primarily developed to assess workers’ sitting times in our previous studies [11, 12]. WLAQ can be used to measure a worker’s time spent sitting within four typical domains of a worker’s life: (a) working time, (b) commuting time, (c) nonworking time on a workday, and (d) time on a non-workday. Matsuo et al. [12] evaluated WLAQ and demonstrated favorable test–retest reliability values and criterion (vs. activPAL) validity values for the four sitting times.

Age, sex, and body fat-related values have often been used in VO_2max_ estimation models [13–15]. Given that measurement accessibilities differ among different body fat-related values such as body mass index (BMI), waist girth (WG), and %fat, these previous studies [13–15] investigated the predictive power for each body fat item. Furthermore, previous studies [14–18] have shown that physical activity (PA) data, such as frequency, duration, and intensity, contribute to the estimation of VO_2max_. However, the original WLAQ can help assess workers’ sitting times but not other PA data. Thus, WLAQ can be modified for evaluating workers’ CRF if additional PA data are collected, and it can be used along with age, sex, and body fat-related values to develop an equation model for VO_2max_ estimation.

Therefore, the purposes of this study were 1) to investigate associations between measured VO_2max_ and sitting times and other PA data collected using the modified WLAQ (m-WLAQ); 2) to investigate criterion validity of a developed equation model for estimating VO_2max_; 3) to compare the accuracy of equation models that include BMI, WG, and %fat; and 4) to investigate the test–retest reliabilities of values derived using m-WLAQ.

## Methods

### Participants

The inclusion criteria were as follows: 1) aged 30–60 years, 2) living in the Tokyo area (Tokyo, Saitama, Chiba, and Kanagawa Prefectures) of Japan, 3) part-time or full-time worker for at least 3 days a week, and 4) no medical conditions that could affect VO_2max_ testing. Participants were recruited through a website advertisement. In total, 202 working adults (97 women and 105 men) participated in this study. The participants visited our laboratory to complete anthropometric measurements, a treadmill exercise test, and m-WLAQ. We excluded 4 participants due to insufficient data for the analyses. Consequently, 198 participants (93 women and 105 men) were included in the validity analysis. Furthermore, among the included participants, 97 participants (42 women and 55 men) visited our laboratory for a second time, with an interval of 1 week between the visits. At the second visit, they completed m-WLAQ again for test–retest reliability analyses.

This study was conducted in accordance with the guidelines proposed in the Declaration of Helsinki. The Ethical Committee of the National Institute of Occupational Safety and Health, Japan reviewed and approved the study protocol (ID H2810). Before obtaining written informed consent, the aims and design of this study were explained to each participant.

### Measures

#### Anthropometric measurements

For each participant, height (to the nearest 0.1 cm) was measured once using a wall-mounted stadiometer (YG-200, Yagami, Nagoya, Japan). Body weight (to the nearest 0.1 kg) and %fat with bioelectrical impedance analysis were assessed using a body composition analyzer (InBody-3.2; Biospace, Seoul, Korea). WG (to the nearest 0.1 cm) was measured twice at the level of the umbilicus in the standing position by a skilled member of the research staff. BMI was calculated as weight (in kilograms) divided by the square of height (in meters).

#### m-WLAQ

m-WLAQ was used to evaluate participants’ sitting times during working time, commuting time, and leisure time on a workday and non-workday. A previous study [12] using the original WLAQ demonstrated favorable test–retest reliability values [intraclass correlation coefficient (*ICC*) = 0.72–0.98] and criterion (activPAL) validity values (Spearman’s *ρ* = 0.40–0.82) for the four sitting times. The original WLAQ was modified to add several questions for collecting PA data (frequency, duration, and intensity) for developing m-WLAQ. The PA score (0–44 points) was calculated as the sum of the points scored for PA data. For its calculation, PA intensity was weighted more heavily than PA duration and frequency on the PA score as per previous studies [17, 19]. An additional PDF file shows the m-WLAQ and calculation method for each value (See Additional file 1).

#### Maximal oxygen consumption

The participants underwent an electrocardiogram-monitored, exhaustion-limited, graded exercise test on a treadmill (EXCITE RUN, Technogym, Cesena, Italy) using the Bruce protocol to determine VO_2max_. During the test, an open-circuit computerized indirect calorimeter (AE-310S, Minato Medical Science, Osaka, Japan) was used to measure ventilation and expired gases. The gas analyzer was calibrated before each trial. Heart rate (HR) was monitored using an electrocardiogram monitor (LifeScope, NIHON KOHDEN, Tokyo, Japan), and a rating of perceived exertion (RPE), using the 6–20 Borg RPE scale, was recorded during the exercise test. The highest 30-s average VO_2_ value was defined as the VO_2max_ value. The exercise test was considered to achieve VO_2max_ when three of the following four criteria were satisfied: 1) respiratory exchange ratio > 1.10, 2) achievement of maximum HR within 10 bpm of the age-predicted maximal (220 − age), 3) RPE > 17, and 4) VO_2_ plateau despite further increases in workload [20, 21].

### Data analysis

Unpaired Student’s t-tests were performed to evaluate differences between groups. Chi-squared tests were used to analyze categorical variables. Pearson’s correlation coefficients were calculated to evaluate the relationship between the measured VO_2max_ and other measurement values. Multiple linear regression analysis was used to develop prediction equations for VO_2max_. Changes in the squared multiple correlation coefficient (*R*^2^) and the standard error of the estimate (*SEE*) were used to assess the incremental gain in variance explained by the different variables added to the model. In the course of previous studies [13–15], *R*^2^ and *SEE* were compared among some types of body fat evaluation models, i.e., BMI, WG, and %fat models, to investigate the influence of differences in methodology, because subjects’ body fat is assessed in several ways in epidemiological surveys. The generated models were cross-validated using the predicted residual error sum of squares (*PRESS*) statistical method [22]. This method calculates the error in prediction for each case when only that case is excluded from generating the estimation model and applying the model to the excluded case. *PRESS* adjusted *R*^2^ (*R*^2^_*p*_) is calculated as 1 − (*PRESS* statistic/SS _total_), whereas *PRESS SEE* (*SEE*_*p*_) is calculated using the following equation: *SEE*_*p*_ = $\sqrt{PRESS\ statistic/N}$. The generated models were further validated by comparing the constant errors (*CEs*) among the subgroups of sex, age, and VO_2max_ levels. *CE* is calculated as the mean difference between measured VO_2max_ and predicted VO_2max_. The test–retest reliability was examined using *ICC* and 95% confidence interval (95% CI) with an *ICC* of < 0.40 indicating *poor* repeatability, 0.40–0.75 indicating *fair-to-good* repeatability, and > 0.75 indicating *excellent* repeatability [23]. *P*-value of < 0.05 was considered statistically significant. All analyses were conducted using SAS, version 9.4 (SAS Institute Japan, Tokyo, Japan).

## Results

Table 1 summarizes the demographic characteristics of the participants. We observed higher anthropometric measurements in men than in women. Although sitting times during worktime and on a non-workday were significantly longer in men than in women, sitting time during leisure time on workday was significantly longer in women than in men. The percentage of clerical jobs was high in both sexes, but the percentages of sales and marketing and engineer/researcher were higher in men.

**Table 1:** Descriptive characteristics of the study participants (*n* = 198)

	Women (*n* = 93)	Men (*n* = 105)	*P*
Age, years	46.7 ± 7.5	47.1 ± 7.1	0.68
Height, cm	158.8 ± 5.2	171.2 ± 5.6	< 0.01
Body weight, kg	54.2 ± 8.1	68.7 ± 9.1	< 0.01
BMI	21.5 ± 2.9	23.4 ± 2.9	< 0.01
WG, cm	75.8 ± 8.9	82.9 ± 8.3	< 0.01
%Fat	29.0 ± 5.6	22.5 ± 5.3	< 0.01
VO_2max_, ml·kg^-1^·min^-1^	35.0 ± 5.5	41.4 ± 6.2	< 0.01
Sitting time obtained using m-WLAQ			
During commuting time, min·d^-1^	12 ± 18	16 ± 21	0.23
During working time, min·d^-1^	382 ± 147	435 ± 162	0.02
During leisure time on workday, min·d^-1^	241 ± 103	183 ± 85	< 0.01
During non-work day, min·d^-1^	513 ± 166	566 ± 192	0.04
Total, min·d^-1^	1148 ± 260	1200 ± 306	0.20
Participants’ occupations, *n* (%)			
Clerical job	62 (66.7)	35 (33.3)	< 0.01
Sales and marketing	19 (20.4)	36 (34.3)	
Driver/Cleaner/Plant worker	1 (1.1)	7 (6.7)	
Medical profession/Teacher	6 (6.5)	4 (3.8)	
Engineer/Researcher	5 (5.4)	23 (21.9)	

Values are presented as mean ± standard deviation or *n* (%). *BMI* body mass index, *m-WLAQ* modified Worker’s Living Activity-time Questionnaire, *VO*_*2max*_ maximal oxygen consumption, *WG* waist girth. *P* values show the results of group difference analyses

Table 2 shows the questions included in m-WLAQ, answer options (and their assigned points), and the results of the study participants. Although the answer trends were different between women and men for Q8 and Q10, the PA score showed no significant sex difference.

**Table 2:** Questions of m-WLAQ for CRF estimation and the results of this study participants (*n* = 198)

Questions		Answer options (points*)	Women (*n* = 93)	Men (*n* = 105)	*P*
Q6	How much breathing-inducing (heart rate increasing) tasks do you perform on an average day during your working hours?	1. none/almost none (0)	81 (87.1)	76 (72.4)	0.07
		2. rarely (3)	7 (7.5)	19 (18.1)	
		3. sometimes (5)	5 (5.4)	9 (8.6)	
		4. often (10)	0 (0.0)	1 (1.0)	
Q8	In your leisure time on a workday, how much intentional physical activity do you engage in?	1. none/almost none (0)	45 (48.4)	54 (51.4)	0.05
		2. 1–3 days a month (1)	6 (6.5)	18 (17.1)	
		3. 1 or 2 days a week (2)	27 (29.0)	18 (17.1)	
		4. ≥3 days a week (3)	15 (16.1)	15 (14.3)	
Q9	If options 2–4 were selected from Q8, please provide the average exercise time per day	1. < 15 min (1)	1 (1.1)	2 (1.9)	0.10
		2. 15–30 min (2)	12 (12.9)	6 (5.7)	
		3. 31–60 min (3)	25 (26.9)	22 (21.0)	
		4. > 60 min (4)	10 (10.8)	21 (20.0)	
		N/A (Q8 = 1)	45 (48.4)	54 (51.4)	
Q10	If options 2–4 were selected from Q8, please tell us the approximate intensity of the exercise per session	1. no sweating or panting (0)	15 (31.3)	4 (3.8)	< 0.01
		2. sweating and panting (3)	29 (60.4)	32 (30.5)	
		3. strained breathing (5)	4 (8.3)	14 (13.3)	
		4. to the point of exhaustion (10)	0 (0.0)	1 (1.0)	
		N/A (Q8 = 1)	45 (48.4)	54 (51.4)	
Q13	On holidays, how much intentional physical activity do you engage in?	1. none/almost none (0)	39 (41.9)	36 (34.3)	0.69
		2. 1–2 days a month (1)	17 (18.3)	23 (21.9)	
		3. once a week (2)	24 (25.8)	32 (30.5)	
		4. ≥2 days a week (3)	13 (14.0)	14 (13.3)	
Q14	If options 2–4 were selected from Q13, please provide the average exercise time per day	1. < 15 min (1)	2 (2.2)	2 (1.9)	0.61
		2. 15–30 min (2)	4 (4.3)	8 (7.6)	
		3. 31–60 mi (3)	27 (29.0)	27 (25.7)	
		4. > 60 min (4)	21 (22.6)	32 (30.5)	
		N/A (Q13 = 1)	39 (41.9)	36 (34.3)	
Q15	If options 2–4 were selected from Q13, please tell us the approximate intensity of the exercise per session	1. no sweating or panting (0)	10 (10.8)	10 (9.5)	0.20
		2. sweating and panting (3)	38 (40.9)	41 (39.0)	
		3. strained breathing (5)	6 (6.5)	17 (16.2)	
		4. to the point of exhaustion (10)	0 (0.0)	1 (1.0)	
		N/A (Q13 = 1)	39 (41.9)	36 (34.3)	
PA score (total points of Q6, Q8, Q9, Q10, Q13, Q14, and Q15/0–44 points)			8.8 ± 7.7	10.7 ± 8.6	0.11

Values are presented as *n* (%) or mean ± standard deviation. *If the respondent selected option #1 on Q8, the scores on Q9 and Q10 should be zero. Similarly, if the respondent selected option #1 on Q13, the scores on Q14 and Q15 should be zero. *CRF* cardiorespiratory fitness, *m-WLAQ* modified Worker’s Living Activity-time Questionnaire. *P* values show the results of group difference analyses

Table 3 shows Pearson’s correlation coefficients between the measured VO_2max_ and other variables. Participants who were older, women, and those with higher body fat-related values had lower VO_2max_. Significant negative correlations were observed between VO_2max_ and sitting time during leisure time on workday (*r* = − 0.27, *P* < 0.01) and total sitting time (*r* = − 0.15, *P* = 0.03), although sitting times during commuting time, working time, and non-workday were not significantly correlated with VO_2max_. Significant positive correlations were observed between PA data (frequency, duration, and intensity) and measured VO_2max_ with the largest association (*r* = 0.47, *P* < 0.01) revealed for the PA score.

**Table 3:** Pearson’s correlation coefficients between measured VO_2max_ and other values (*n* = 198)

	Age (years)	Sex women:0, men:1	BMI	WG (cm)	%fat	Sitting time (min·d^−1^)				
						Commuting time	Working time	Leisure time on workday	Non-workday	Total
*r*	−0.29	0.48	−0.20	−0.24	− 0.64	−0.11	− 0.02	−0.27	− 0.07	−0.15
*P*	< 0.01	< 0.01	< 0.01	< 0.01	< 0.01	0.14	0.79	< 0.01	0.36	0.03
	Working time	Leisure time on workday (points)			Non-workday (points)			PA score (points)		
	PA intensity (points)	Exercise frequency	Exercise duration	Exercise intensity	Exercise frequency	Exercise duration	Exercise intensity			
*r*	0.22	0.29	0.35	0.42	0.31	0.36	0.43	0.47		
*P*	< 0.01	< 0.01	< 0.01	< 0.01	< 0.01	< 0.01	< 0.01	< 0.01		

*BMI* body mass index, *PA* physical activity, *VO*_*2max*_ maximal oxygen consumption, *WG* waist girth. Correlation coefficient (r) and its *P* value are displayed

The variables significantly related to the measured VO_2max_ were included as predictors in multiple regression models (Table 4). The model that included age, sex, and BMI accounted for 43% of the variance in measured VO_2max_ (adjusted *R*^2^ = 0.43, *SEE* = 5.04 ml·kg^− 1^·min^− 1^ or 13.1%). When the PA score obtained using m-WLAQ was added to the model that included age, sex, and BMI, the adjusted *R*^2^ significantly improved by 16% (adjusted *R*^2^ = 0.59, *SEE* = 4.29 ml·kg^− 1^·min^− 1^ or 11.2%). Similarly, regarding the models using WG or %fat instead of BMI, addition of the PA score improved the variance of the models by 12% or 11%, respectively. The cross-validation results from the *PRESS* method are also shown in Table 4. The decrease in *R*^2^ (approximately 0.01) and increase in *SEE* value (approximately 0.05 ml·kg^− 1^·min^− 1^) were small for all three models.

**Table 4:** Regression coefficients for prediction of VO_2max_ using selected independent values (*n* = 198)

	Intercept	Independent variables						Adjusted R^2^	*SEE*		Cross-validation	
		Age	Sex	BMI	WG	%fat	PA score				*R*_*2*_*p*	*SEEp*
		yrs	women:0men:1		cm	%	points		ml·kg^−1^·min^−1^	%		ml·kg^−1^·min^−1^
BMI model	63.90	−0.25	8.02	−0.79	–	–	–	0.43	5.04	13.1	0.42	5.08
	59.96	−0.23	7.39	−0.79	–	–	0.33	0.59	4.29	11.2	0.58	4.33
WG model	70.39	−0.22	8.82	–	−0.33	–	–	0.49	4.78	12.5	0.48	4.82
	64.70	−0.21	8.02	–	−0.29	–	0.29	0.61	4.17	10.9	0.60	4.22
%fat model	62.45	−0.25	2.95	–	–	−0.54	–	0.51	4.69	12.2	0.49	4.74
	57.50	−0.24	2.83	–	–	−0.48	0.28	0.62	4.13	10.8	0.61	4.18

*PA* physical activity, *R*^*2*^*p* PRESS R^2^, *SEE* standard error of estimate, *SEE p* PRESS SEE, *VO*_*2max*_ maximal oxygen consumption, *WG* waist girth; −, not included in the model

Table 5 presents the results of other cross-validation analyses using *CE* values. The absolute *CE* values for the sex and age subgroups were < 1.00 for all three models. Regarding the VO_2max_ subgroups, *CE* values were negatively high (overestimation) for the low-fitness subgroup and positively high (underestimation) for the high-fitness subgroup, whereas lower *CE* values were observed for the mid-fitness subgroup in all three models.

**Table 5:** Constant error for subgroups of sex, age, and measured VO_2max_ (*n* = 198)

	N (%)	Measured VO_2max_	BMI model*		WG model*		%fat model*	
			Predicted VO_2max_	CE	Predicted VO_2max_	CE	Predicted VO_2max_	CE
Sex								
Women	93 (47.0)	35.0 ± 5.5	35.2 ± 4.0	−0.17 ± 4.22	35.5 ± 4.1	−0.48 ± 4.17	34.9 ± 4.3	0.16 ± 3.92
Men	105 (53.0)	41.4 ± 6.2	41.6 ± 4.2	−0.18 ± 4.29	41.9 ± 4.2	−0.51 ± 4.11	41.3 ± 4.2	0.13 ± 4.24
Age								
30–39	29 (14.7)	41.4 ± 6.0	42.4 ± 4.1	−0.91 ± 3.75	42.4 ± 4.5	−0.98 ± 3.59	41.8 ± 3.9	−0.39 ± 3.76
40–49	88 (44.4)	39.1 ± 7.3	38.8 ± 5.4	0.32 ± 4.52	39.3 ± 5.4	−0.12 ± 4.49	38.7 ± 5.6	0.40 ± 4.35
50–59	81 (40.9)	36.5 ± 5.6	36.9 ± 4.5	−0.45 ± 4.08	37.2 ± 4.6	−0.73 ± 3.92	36.4 ± 4.7	0.05 ± 3.92
VO_2max_								
Low	66 (33.3)	31.4 ± 2.6	34.1 ± 3.7	−2.64 ± 2.88	34.2 ± 3.7	−2.80 ± 2.89	33.5 ± 3.6	−2.07 ± 2.87
Middle	66 (33.3)	37.7 ± 1.6	38.6 ± 3.5	− 0.91 ± 3.36	39.0 ± 3.6	−1.27 ± 3.45	38.3 ± 3.7	− 0.58 ± 3.60
High	66 (33.3)	46.0 ± 4.2	43.0 ± 3.8	3.02 ± 4.23	43.4 ± 3.6	2.58 ± 3.99	42.9 ± 3.7	3.08 ± 3.87

*BMI* body mass index, *CE* constant error, *VO*_*2max*_ maximal oxygen consumption, *WG* waist girth

* Each model includes age, sex, and PA score in addition to the titled values (BMI, WG, and %fat) as independent variables

The test–retest reliabilities were examined using the data of the 97 participants who completed m-WLAQ twice. Table 6 shows the *ICC* of sitting times and PA-related values obtained using m-WLAQ. The *ICC* values for sitting times were *fair to good* (commuting time and non-workday) or *excellent* (working time and leisure time on workday and total sitting time). All *ICC* values of PA-related values were *excellent* except those for exercise duration on non-workday and exercise intensity during leisure time on workday and non-workday, whose values were *fair to good*. The *ICC* value of the PA score was 0.87, indicating *excellent* reliability.

**Table 6:** Test–retest reliability of sitting times and PA-related values measured using data obtained with m-WLAQ at sitting times 1 and 2 (*n* = 97)

	*ICC*	95%CI
Sittting time		
Commuting time	0.67	0.54–0.77
Working time	0.94	0.91–0.96
Leisure time on workday	0.79	0.70–0.85
Non-workday	0.66	0.53–0.76
Total	0.81	0.73–0.87
Exercise frequency		
Leisure time on workday	0.78	0.69–0.85
Non-workday	0.88	0.83–0.92
Exercise duration		
Leisure time on workday	0.82	0.68–0.90
Non-workday	0.71	0.55–0.82
PA or Exercise intensity		
Working time	0.75	0.65–0.83
Leisure time on workday	0.55	0.29–0.73
Non-workday	0.48	0.25–0.66
PA score	0.87	0.82–0.91

*CI* confidence interval, *ICC* intraclass correlation coefficient, *PA* physical activity, *m-WLAQ* modified Worker’s Living Activity-time Questionnaire

**Fig. 1:**
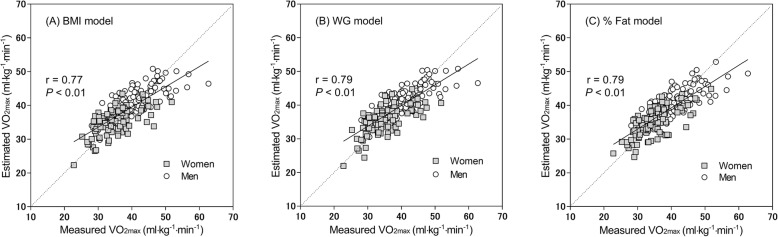
Relationship between measured and estimated VO_2max_ in (A) BMI, (B) WG, and (C) %fat models. The regression line and correlation coefficient with *P* values are displayed. The dashed lines are the identity lines of the measured and estimated VO_2max_.

The relationships between measured VO_2max_ and estimated VO_2max_ are shown in Fig. 1. Estimated VO_2max_ correlated well with measured VO_2max_ for all three body fat models; Fig. 1 also shows overestimations in participants with low fitness and underestimations in participants with high fitness for all three models.

## Discussion

The study showed that 1) the PA score obtained using m-WLAQ, rather than sitting times, was associated with measured VO_2max_; 2) the equation models that included age, sex, body fat-related values, and PA score obtained using m-WLAQ had favorable validity for estimating VO_2max_; 3) no appreciable difference was observed in estimated VO_2max_ among the three models with regard to BMI, WG, and %fat; and 4) favorable reliability values were shown for sitting times and the PA score obtained using m-WLAQ.

Consistent with the findings of a previous study [24], significant negative correlations were observed between sitting times and measured VO_2max_ (Table 3). However, sitting times were not accepted as an effective explanatory variable for estimating VO_2max_ in our regression analyses. In contrast, questionnaire-based PA data, such as frequency, duration, and intensity, were significantly correlated with measured VO_2max_ (Table 3), and the regression model identified the PA score to be the principal explanatory value for the equation models. The PA score was calculated for precise VO_2max_ prediction in reference to the HUNT study by Nes et al. [17] and a previous exercise intervention study [19]. Nes at al [17]. used some question items regarding PA frequency, duration, and intensity for estimating VO_2max_ and relative weightings of different responses were set on the basis of their relation to VO_2max_. In their estimation, PA intensity was weighted more heavily than PA duration and frequency on the PA score. Further, an exercise intervention study [19] emphasized the primacy of PA intensity rather than PA duration and volume in improving VO_2max_. We followed these studies to develop the PA score, i.e., the questions regarding intensity, such as Q6, Q10, and Q15, were weighted more heavily than other questions (Table 2). The PA score was strongly correlated with VO_2max_ (Table 3) and functioned well for estimating VO_2max_ (Table 4). The results of previous studies and the present study suggest that PA intensity can have a potential role in estimating VO_2max_.

Age, sex, and body fat-related values were significantly correlated with measured VO_2max_ (Table 3), and these three factors accounted for 43–51% of the variance in measured VO_2max_ (Table 4). These percentages increased by 11–16% following to addition of the PA score obtained using the questionnaire in the model (Table 4). Jackson et al. [14] suggested questionnaire-based VO_2max_ prediction models including age, sex, body fat-related values, and the PA score obtained using the questionnaire, demonstrating *SEE*s of 5.35–5.70 ml·kg^− 1^·min^− 1^. Similarly, Wier et al. [15] suggested questionnaire-based VO_2max_ prediction models including age, sex, body fat-related values, and the PA score obtained using the questionnaire, demonstrating *SEE*s of 4.72–4.90 ml·kg^− 1^·min^− 1^. Furthermore, Malek et al. [25] developed a VO_2max_ prediction equation including age, body weight, height, and questionnaire-based exercise values, which had an *SEE* value of 4.12 ml·kg^− 1^·min^− 1^. The present study showed results similar to these previous studies, with prediction model *SEE*s of 4.13–4.29 ml·kg^− 1^·min^− 1^. These *SEE* values can be replaced with %*SEE* values of 10.8–11.2%. Other types of VO_2max_ estimation studies reported %*SEE* values of 11.4% in the 20-m shuttle run test study [26] and 10–15% in wearable device studies [13, 27, 28]. The *SEE* values in the present study seem to be favorable when compared with those calculated in other VO_2max_ prediction studies.

Regarding the method to validate a regression equation, although the data-splitting method is well known, in which the entire data are divided into a fitting group and validation group, the *PRESS* method [22] is particularly recommended for studies with a small sample size. This method can provide useful diagnostics while avoiding the disadvantages of the data-splitting method such as lack of equation stability due to diluted sample size. In fact, studies with a large sample size, such as those of Jackson et al. (1999 participants) [14] and Nes at al. (4637 participants) [17] used the data-splitting method. However, the *PRESS* method has not only been used in studies with a large sample size such as in that of Matthews et al. (799 participants) [29] and Wier at al. (2801 participants) [15] but also in studies with a small sample size such as those of Malek et al. (115 participants) [25] and Cao et al. (148 participants) [13]. The *PRESS* method appeared to be appropriate for the present study on 198 participants.

Jackson et al. [14] recommended questionnaire-based VO_2max_ prediction models including age, sex, the PA score obtained using the questionnaire, and body fat-related values such as %fat (skinfold method) and BMI, and they demonstrated *SEE* values of 5.35 ml·kg^− 1^·min^− 1^ for the %fat model and 5.70 ml·kg^− 1^·min^− 1^ for the BMI model. Wier et al. [15] also recommended questionnaire-based VO_2max_ prediction models including age, sex, the PA score obtained using the questionnaire, and body fat-related values such as %fat (skinfold method), WG, and BMI and they showed no considerable differences in accuracy among the three models using WG (*SEE* value of 4.80 ml·kg^− 1^·min^− 1^), %fat (*SEE* value of 4.72 ml·kg^− 1^·min^− 1^), or BMI (*SEE* value of 4.90 ml·kg^− 1^·min^− 1^). The present study obtained results similar to those of previous studies, i.e., no considerable difference was observed in accuracy among the three body fat-related variables, i.e., BMI, WG, and %fat (bioelectrical impedance analysis). Although the *SEE* value of the BMI model (4.29 ml·kg^− 1^·min^− 1^ or 11.2%) was relatively higher than those of the WG (4.17 ml·kg^− 1^·min^− 1^ or 10.9%) and %fat (4.13 ml·kg^− 1^·min^− 1^ or 10.8%) models, which are consistent with the findings reported by Wier et al. [15], the BMI model could be more convenient than the other models because BMI is a basic and less burdensome assessment item in adult health checkups. Therefore, the following equation model is suggested for VO_2max_ estimation in the present study (using sex = 0 for women and 1 for men): VO_2max_ = 59.96 + (− 0.23 × age) + (7.39 × sex) + (− 0.79 × BMI) + (0.33 × PA score).

There are some limitations to the present study. First, response bias may have occurred because the participants had advance knowledge of the experimental procedure, i.e., they could decide to participate in this study after viewing our research advertisement, which may have led to greater inclusion of participants preferring PA or exercise. Second, *CE* analyses (Table 5) and scatter graphs (Fig. 1) showed that the CRF evaluation model derived in the present study significantly underestimated VO_2max_ in participants with high fitness and overestimated VO_2max_ in those with low fitness. This systematic error usually occurs in VO_2max_ estimation studies [15, 17]. As pointed out by other researchers [15, 17], while underestimation in individuals with high fitness may not be a pressing problem because high fitness relates to low disease and mortality risks, overestimation in individuals with low fitness may be more problematic because low fitness relates to increasing disease risks. A correction method such as a compensation formula or including a convenient stress test should be considered to correct the error, particularly in individuals with low fitness. Third, we could not include approximately 50% of the participants in test–retest reliability analyses because they did not participate in the second round of m-WLAQ. Participant selection bias could have occurred because the selection was not conducted at random but in accordance with participant convenience. Fourth, in recent public health research, moderate-to-vigorous intensity PA (MVPA) has been treated as an important terminology separately from SB [30, 31]. MVPA and SB are defined as accelerometry-measured PA of ≥3.0 metabolic equivalents (METs) and PA of ≤1.5 METs, respectively [32]. m-WLAQ can assess SB but not MVPA.

## Conclusion

The PA score obtained using m-WLAQ, rather than sitting time, correlated well with measured VO_2max_ and had a favorable test–retest reliability. The equation model that included PA score with age, sex, and BMI had a favorable validity for estimating VO_2max_. Thus, the study suggests that m-WLAQ can be a useful questionnaire to assess workers’ CRF and SB.

The findings of this study could help to advance the quality of future epidemiological surveys in occupational health research fields. On the other hand, given the CRF classification based only on age, sex, BMI, and questionnaire results could impose a limitation for detecting differences among individuals, further research is necessary to advance CRF assessment. Use of sophisticated wearable sensors could improve classification [33].

## Supplementary information

**Additional file 1.** The modified Worker’s Living Activity-time Questionnaire (m-WLAQ)

## Data Availability

Raw data were generated at the Research Center for Overwork-Related Disorders (RECORD) (https://www.jniosh.johas.go.jp/en/groups/overwork.html). On reasonable request, derived data supporting the findings of this study are available from the corresponding author, TM, after approval from RECORD and the Research Ethics Committee.

## Abbreviations

BMI: Body mass index
CE: Constant error
CRF: Cardiorespiratory fitness
ICC: Intraclass correlation coefficients
m-WLAQ: modified Worker’s Living Activity-time Questionnaire
PA: Physical activity
PRESS: Predicted residual sum of squares
SB: Sedentary behavior
SEE: Standard error of estimate
VO_2max_: Maximal oxygen consumption
WG: Waist girth
