# Supplementary material for: Workers’ physical activity data contribute to estimating maximal oxygen consumption: a questionnaire study to concurrently assess workers’ sedentary behavior and cardiorespiratory fitness
Source: BMC Public Health. 2020 Jan 8;20:22. doi: 10.1186/s12889-019-8067-4 (PMC6950791; doi:10.1186/s12889-019-8067-4)
Supplement: Supplementary file 1 — Additional file 1. The modified Worker’s Living Activity-time Questionnaire (m-WLAQ) [file 12889_2019_8067_MOESM1_ESM.docx]

You will be asked about your “**workday**.” Please try to recall the events of the past month or so, and try to imagine how your “**average workday**” was.

**Q1. On the day before a workday** (includes going to bed after midnight), when is your **bedtime**? This is defined as the time when you get into bed (such as lying down on a bed) and not the time of falling asleep. Additionally, at what time do you **wake up on a workday**? This is defined as the time you actually get out of bed as opposed to the time when you wake up.

**Bedtime on the day before a workday　　　　　　　　　 Wake-up time on workdays**

|  |  | h |  |  | min |  |  | h |  |  | min |
| --- | --- | --- | --- | --- | --- | --- | --- | --- | --- | --- | --- |

(24-h notation) (24-h notation)

**Q2.** **On a working day,** at what time do you **leave home** and at what time do you **arrive at work**? If you are a full-time housewife, use your home as your workplace and do not commute, check a box ✓ next to “No commute” and mention the time when you start your work.

**Time you leave home Time you arrive at work (morning/afternoon)**

|  |  | h |  |  | min |  |  | h |  |  | min |
| --- | --- | --- | --- | --- | --- | --- | --- | --- | --- | --- | --- |

(24-h notation) (24-h notation)

**No commute ( ) ⇒** work start time **( )** (h) **( )** (min) (24-h notation)

**Q3**. Regarding the **one-way commuting time (duration between departing home and arriving at work)** in Q2, which of the following transportation methods do you use, and how long do you spend in each mode of transportation? Even if commuting time differs depending on the day, please answer based on an average day.

A) Time spent **on foot** (including running) or on a bicycle ( ) min

B) Time spent **sitting** in a car, motorbike, train, bus/at a station, etc. ( ) min

C) Time spent **standing** on a train, bus/at a station, etc. ( ) min

D) Other time ( ) min

* The total of one-way commuting time should be the sum of A to D.

**Q4.** At what time do you usually **leave your workplace** after finishing work on a workday**?**

|  |  | h |  |  | min |
| --- | --- | --- | --- | --- | --- |

(24-h notation)

**Q5.** During the **working hours** of an average day (excluding commuting time), what do you think is **the percentage of time you spend (1) sitting vs. (2) standing or walking**? ‘Sitting time’ is defined as desk work, meetings, travel time by car, motorbike, train, and bus; and at a station. ‘Time spent standing or walking’ refers to time on foot or use of a bicycle. Please consider the sum of both the times for totaling up to 100%.

1. Time spent sitting ( )%
2. Time spent standing or walking ( )%

**Q6.** How much **breathing-inducing (heart rate increasing) tasks** do you perform on an average day **during your working hours** (excluding commuting time)? Please select one answer from 1 to 4.

1. none/almost none　　2) rarely　　3) sometimes　　4) often

With regard to free time on workdays

**Q7.** Regarding leisure time excluding “sleep,” “commuting,” and “work,” (your free time after work, time spent on doing housework, leisure time at home, etc.) **on a workday**, select the description from 1 to 4 that best describes your situation.

Note) Time spent in cars and motorbikes is considered “sitting time,” whereas time spent riding a bicycle or exercising is considered “time spent standing or walking.”

1) Most time is spent sitting or lying down 2) More time is spent sitting or lying down

3) More time is spent standing or walking 4) Most time is spent standing or walking

**Q8.** In your leisure time on a workday (such as leisure after work or time at home), **how much intentional physical activity** do you engage in (i.e., walking, jogging, going to the gym, playing in a sports team or at school, etc.)? Please select one option that best describes your situation.

1) none/almost none 2) 1–3 days a month 3) 1 or 2 days a week 4) ≥3 days a week

**Q9.** If options 2–4 were selected from Q8, please provide **the average exercise time** per day.

1) <15 min 2) 15–30 min 3) 31–60 min 4) >60 min

**Q10.** If options 2–4 were selected from Q8, please tell us **the approximate intensity of the exercise** per session.

1) No sweating or panting

2) Sweating and panting (heart rate increases)

3) Strained breathing

4) To the point of exhaustion

**→Next page**

The questions below concern **holidays** (i.e., days without work). Please recall the events of the past month or so, and try to imagine how your “**average holiday**” was.

**Q11.** When is your **bedtime** (includes going to bed after midnight) **the day before a holiday**? This is defined as the time when you get into bed (such as lying down on a bed) and not the time of falling asleep. Additionally, at what time do you **wake up on a holiday**? This is defined as the time you actually get out of bed as opposed to the time when you wake up.

**Bedtime the day before a holiday Wake-up time on holiday**

|  |  | h |  |  | min |  |  | h |  |  | min |
| --- | --- | --- | --- | --- | --- | --- | --- | --- | --- | --- | --- |

(24-h notation) (24-h notation)

**Q12.** Excluding the time spent sleeping on a holiday, what do you think is **the ratio of the time you spend sitting or lying down vs. standing or walking**? Please consider the sum of both the times for totaling up to 100%.

1. Time spent sitting or lying down ( )%
2. Time spent standing or walking ( )%

**Q13.** On holidays, **how much intentional physical activity** do you engage in (i.e., walking, jogging, going to the gym, playing on a sports team or schools, etc.)? Please select one option that best describes your situation.

1) none/almost none 2) 1–2 days a month 3) Once a week 4) ≥2 days a week

**Q14.** If options 2–4 were selected from Q13, please provide **the average exercise time** per day

1) <15 min 2) 15–30 min 3) 31–60 min 4) >60 min

**Q15.** If options 2–4 were selected from Q13, please tell us **the approximate intensity of the exercise** per session.

1) No sweating or panting

2) Sweating and panting (heart rate increases)

3) Strained breathing

4) To the point of exhaustion

**Thank you for your time.**

**Calculations**

- Sitting time during commuting time: Q3B (min)
- Sitting time during working time: total working time (min, in Q2 and Q4) × proportion of sitting time (%, in Q5A)
- Sitting time during free time on a workday: [1440 min − sleeping time (min, in Q1) − working time (min, in Q2 and Q4) − 2 × commuting time (min, in Q2)] × proportion of sitting time (in Q7, option #1: 90%, #2: 60%, #3: 40%, and #4: 10%)
- Sitting time on a nonwork day: [1440 min − sleeping time (min, in Q11)] × proportion of sitting time (%, in Q12A)
- PA score (0–44 points) = Q6 (option #1: 0 points, #2: 3 points, #3: 5 points, and #4: 10 points) + Q8 (#1: 0 points, #2: 1 point, #3: 2 points, and #4: 3 points) + Q9 (#1: 1 point, #2: 2 points, #3: 3 points, and #4: 4 points) + Q10 (#1: 0 points, #2: 3 points, #3: 5 points, and #4: 10 points) + Q13 (#1: 0 points, #2: 1 point, #3: 2 points, and #4: 3 points) + Q14 (#1: 1 point, #2: 2 points, #3: 3 points, and #4: 4 points) + Q15 (#1: 0 points, #2: 3 points, #3: 5 points, and #4: 10 points). If the respondent selected option #1 in Q8, the scores for Q9 and Q10 should be 0. Similarly, if the respondent selected option #1 in Q13, the scores for Q14 and Q15 should be 0.
